# AAV2.7m8-Mediated MicroRNA Expression Suppresses VEGF-Induced Angiogenic Responses in HUVEC

**DOI:** 10.3390/ijms27073123

**Published:** 2026-03-30

**Authors:** Jin Young Yang, Jun-Sub Choi, Tae Kwann Park

**Affiliations:** 1 Laboratory for Translational Research on Retinal and Macular Degeneration, Soonchunhyang University Hospital Bucheon, Bucheon 14584, Republic of Korea; roswellgirl111@gmail.com (J.Y.Y.); mjschoi69@gmail.com (J.-S.C.); 2 Department of Ophthalmology, Soonchunhyang University Hospital Bucheon, Bucheon 14584, Republic of Korea; 3 Department of Interdisciplinary Program in Biomedical Science, Soonchunhyang Graduate School, Soonchunhyang University Hospital Bucheon, Bucheon 14584, Republic of Korea

**Keywords:** angiogenesis, VEGF, AAV2.7m8, MicroRNA, MAPK signaling, HUVEC, ocular gene therapy, retinal neovascularization

## Abstract

Vascular endothelial growth factor (VEGF)-driven pathological angiogenesis constitutes a primary driver of neovascular diseases, including neovascular age-related macular degeneration (nAMD) and diabetic retinopathy (DR). Although anti-VEGF agents demonstrate clinical efficacy, their limited intraocular half-life mandates repeated intravitreal injections, thereby highlighting the imperative for long-term therapeutic strategies. In the present study, we assessed the anti-angiogenic potential of retinal organoid-derived microRNAs (miRNA) delivered via an engineered adeno-associated virus vector. Human umbilical vein endothelial cells (HUVEC) were transduced with AAV2.7m8 vectors to overexpress three candidate miRNA (miR-26a, miR-122, and let-7a), followed by VEGF stimulation to evaluate downstream signaling pathways and angiogenic responses. AAV2.7m8-mediated transduction of HUVEC demonstrated high efficiency without inducing detectable cytotoxicity. Overexpression of these miRNA markedly attenuated VEGF-induced phosphorylation of extracellular signal-regulated kinase (ERK), c-Jun N-terminal kinase (JNK), and p38 MAPK. Functional assays demonstrated suppression of endothelial cell proliferation and cell cycle progression, with miR-122-5p additionally inhibiting migration. All three miRNA substantially inhibited capillary-like tube formation. In aggregate, these results affirm that AAV2.7m8-mediated delivery of retinal organoid-derived miRNA —namely miR-26a-5p, miR-122-5p, and let-7a-5p—markedly suppresses VEGF-induced angiogenic signaling cascades and endothelial cell activation in vitro, thereby establishing their viability as a sustained therapeutic approach for pathological retinal neovascularization.

## 1. Introduction

Vascular endothelial growth factor A (VEGF-A), together with the broader VEGF family of signaling pathways, constitutes a fundamental regulatory axis of angiogenesis and neovascularization. These processes are critically involved in the pathophysiology of chronic inflammation, tumor progression, cardiovascular disease, and ocular disorders. Aberrant or pathological angiogenesis represents a defining feature of vision-threatening retinal diseases, most notably neovascular age-related macular degeneration (nAMD) and diabetic retinopathy (DR) [1,2]. Although anti-vascular endothelial growth factor (VEGF) therapies have become the standard of care, limitations such as the requirement for frequent intravitreal injections and suboptimal responses in some patients underscore the need for more durable and efficacious therapeutic alternatives [3,4,5]. Angiogenesis constitutes a precisely orchestrated, multistep cascade encompassing endothelial cell activation, migration, proliferation, tubulogenesis, and subsequent vascular maturation. In pathological contexts, dysregulated orchestration of these processes culminates in the emergence of aberrant, permeable neovascular networks [6,7,8,9]. Among the signaling cascades driving these processes, VEGF-induced activation of mitogen-activated protein kinase (MAPK) pathways—including extracellular signal-regulated kinase (ERK), c-Jun N-terminal kinase (JNK), and p38 MAPK—serves a central role in mediating initial endothelial cell activation as well as subsequent proliferation, migration, and tube formation in pathological angiogenesis [10,11,12].

Extracellular vesicles (EV) serve as pivotal mediators of intercellular communication by transporting microRNAs (miRNA) that regulate intricate angiogenic signaling pathways [13,14,15]. In contrast to conventional EV, retinal organoid-derived exosomes (RO-Exo) harbor a distinctive repertoire of retina-specific bioactive molecules [16,17]. Recent investigations by our group have further delineated the therapeutic potential of these exosomes [18]. We have previously demonstrated that RO-Exo significantly attenuate choroidal neovascularization (CNV) lesion size and restore retinal pigment epithelium (RPE) integrity in a murine laser-induced CNV model, thereby confirming their in vivo efficacy in suppressing pathological neovascularization [19]. Furthermore, we have previously identified that miR-26a-5p, miR-122-5p, and let-7 family members within these exosomes play a pivotal role in preventing photoreceptor degeneration by modulating key intracellular signaling pathways, including the MAPK pathway [18]. Although these miRNAs were initially characterized for their neuroprotective effects, they may also modulate angiogenic responses in endothelial cells, given the central role of MAPK signaling in VEGF-mediated endothelial activation [20,21,22,23,24]. Moreover, miR-26a has been shown to directly modulate both pathological and physiological angiogenesis, underscoring its pivotal role as a key regulator of angiogenic processes [25,26].

Building upon these prior findings, the present study examines the capacity of these miRNAs to suppress VEGF-induced angiogenic responses in human umbilical vein endothelial cells (HUVEC). HUVEC are a long-established and widely utilized in vitro model in vascular biology, providing a reliable and reproducible platform for investigating endothelial function. As VEGF-mediated signaling represents a central mechanism of pathological angiogenesis, HUVEC serve as a suitable and well-validated model system for studying VEGF-driven endothelial responses across diverse vascular contexts [27,28,29].

Despite their promising therapeutic potential, the clinical utility of native exosomes remains constrained by their short half-life and instability in physiological environments [30,31]. To achieve durable therapeutic effects, we employed the AAV2.7m8 vector system for sustained miRNA delivery [32]. In this study, we investigated whether AAV-mediated overexpression of candidate miRNAs (miR-26a-5p, miR-122-5p, and let-7a-5p) modulates VEGF-induced MAPK activation and angiogenic responses in endothelial cells in vitro.

## 2. Results

### 2.1. Development of an Efficient AAV2.7m8-Mediated miRNA Delivery System and Bioinformatics-Based Functional Prediction

**Figure 1:**
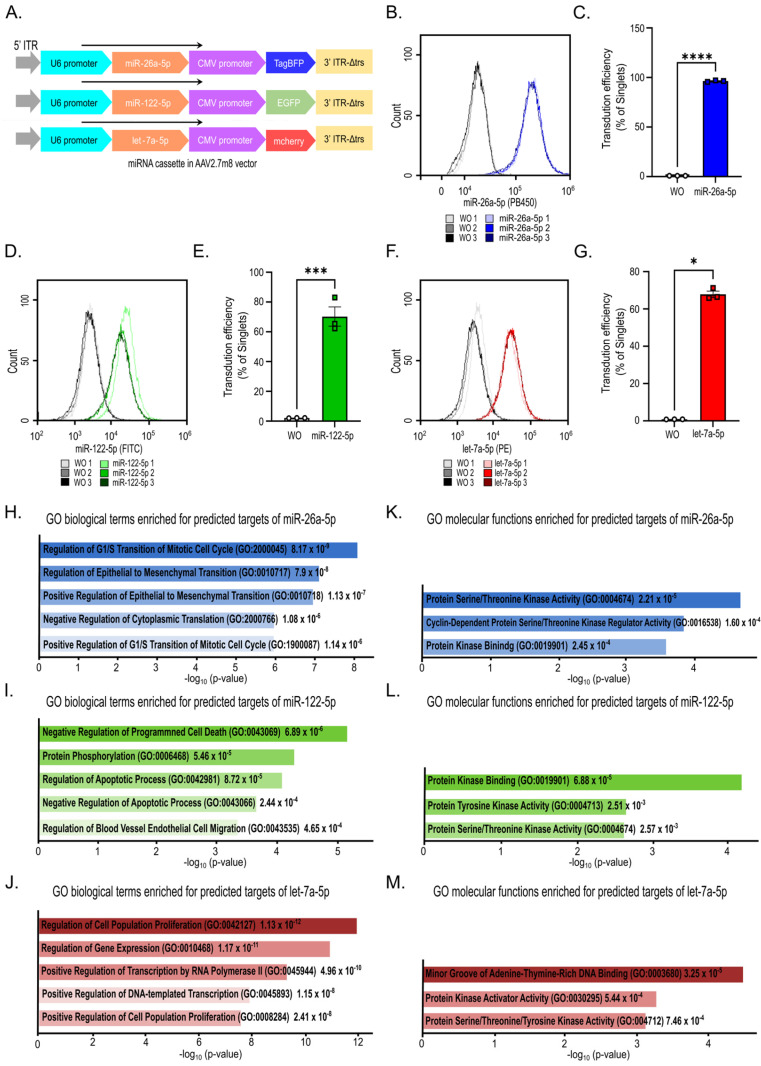
Characterization of the AAV-mediated microRNAs (miRNA) delivery system and bioinformatic prediction of miRNA target functions. (**A**) Schematic representation of AAV2.7m8 vectors expressing miR-26a-5p (TagBFP), miR-122-5p (EGFP), and let-7a-5p (mCherry) under the control of a U6 promoter. Black arrows indicate the transcriptional orientation of each expression cassette, and colors indicate the corresponding reporter genes for each miRNA construct. (**B**,**D**,**F**) Representative flow cytometry histograms showing reporter fluorescence intensity in Human umbilical vein endothelial cells (HUVEC) 24 h after AAV-mediated delivery. Cells were sequentially gated to exclude debris and doublets (using FSC-A/SSC-A and FSC-A/FSC-H plots) to ensure the analysis of a pure singlet population. Non-transduced cells (WO; without AAV treatment) are shown as controls. (**C**,**E**,**G**) Quantification of transduction efficiency, expressed as the percentage of reporter-positive cells within the singlet population. Fluorescence-positive gates were established based on the corresponding non-transduced control. Data are presented as mean ± SEM from three independent experiments (n = 3). Statistical significance was determined using an unpaired Student’s *t*-test. (**H**–**J**) Gene Ontology (GO) biological process (BP) enrichment analysis of experimentally validated miRNA target genes retrieved from miRTarBase for miR-26a-5p, miR-122-5p, and let-7a-5p, highlighting pathways related to cell cycle regulation, apoptosis, endothelial migration, and transcriptional control. (**K**–**M**) GO molecular function (MF) enrichment analysis of experimentally validated miRNA target genes retrieved from miRTarBase for miR-26a-5p, miR-122-5p, and let-7a-5p, showing enrichment for protein kinase activity, kinase binding, and DNA-binding-related functions. Statistical significance is indicated as * *p* < 0.05, *** *p* < 0.005, and **** *p* < 0.001.

To achieve efficient delivery of the three principal miRNAs identified in RO-Exo, we developed an AAV2.7m8-based miRNA expression system (Figure 1A). This AAV2.7m8-based miRNA expression system incorporates distinct fluorescent reporters—TagBFP, EGFP, and mCherry—facilitating direct visualization and quantitative evaluation of transduction efficiency in HUVEC (Figure 1A). HUVEC were transduced with individual AAV vectors and subjected to flow cytometric analysis 24 h post-transduction. All miRNA-expressing AAV vectors exhibited high transduction efficiency (Figure 1B,D,F). In particular, miR-26a-5p achieved a transduction efficiency of 96.38 ± 0.43% (mean ± SEM.; *p* < 0.001; Figure 1B,C). In comparison, miR-122-5p and let-7a-5p achieved transduction efficiencies of 70.23 ± 6.45% and 67.84 ± 1.71%, respectively (Figure 1D–G). These findings demonstrate that the AAV2.7m8 system enables efficient miRNA delivery, well-suited for subsequent functional assays in endothelial cells.

To elucidate the putative biological functions of these miRNAs, Gene Ontology (GO) enrichment analysis was conducted on their experimentally validated target genes retrieved from miRTarBase. Analysis of biological processes (BP) demonstrated significant enrichment of GO terms related to cell cycle regulation, endothelial cell migration, and cell population proliferation among the target genes of three miRNAs (Figure 1H–J). Additionally, molecular functions (MF) analysis revealed consistent enrichment of GO categories associated with protein kinase activity and protein binding (Figure 1K–M). Collectively, these bioinformatics analyses indicate that the target genes of miR-26a-5p, miR-122-5p, and let-7a-5p are intimately associated with endothelial cell activation and kinase-mediated signaling pathways, thereby furnishing a conceptual framework for subsequent interrogation of VEGF-induced angiogenic signaling.

### 2.2. VEGF Enhances HUVEC Viability and Elicits Rapid Activation of Multiple MAPK Signaling Pathways

**Figure 2:**
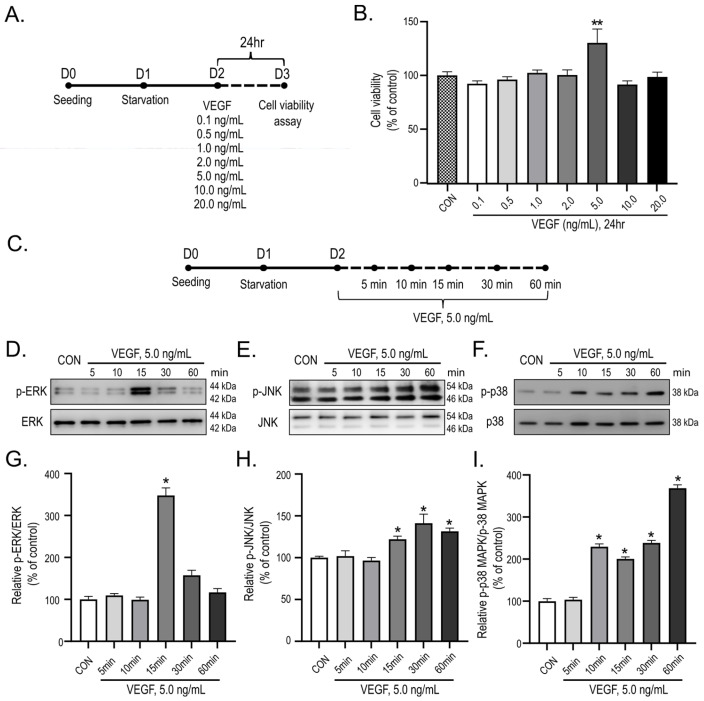
Establishment of vascular endothelial growth factor (VEGF) stimulation conditions and mitogen-activated protein kinase (MAPK) signaling kinetics in HUVEC. In the experimental schematic panels (**A**,**C**), D followed by a number indicates the experimental day, and the dashed line represents the duration of VEGF treatment. (**A**,**B**) Dose-dependent effects of VEGF (0.1–20.0 ng/mL) on HUVEC viability at 24 h following serum starvation. Based on these results, 5.0 ng/mL VEGF was selected for subsequent experiments. (**C**) Experimental schematic illustrating the time course of VEGF stimulation (5.0 ng/mL) for MAPK signaling analysis. (**D**–**F**) Representative Western blot images showing time-dependent phosphorylation of extracellular signal-regulated kinase (ERK), c-Jun N-terminal kinase (JNK), and p38 MAPK following VEGF stimulation for the indicated durations. Total ERK, JNK, and p38 levels were used for normalization. (**G**–**I**) Densitometric quantification of phosphorylated ERK (p-ERK), JNK (p-JNK), and p38 (p-p38) normalized to their total protein levels and expressed relative to the control. Distinct activation kinetics were observed for each MAPK pathway, with p-ERK peaking at 15 min, p-JNK at 30 min, and p-p38 at 60 min. Data are presented as mean ± SEM from three independent experiments (n = 3). Statistical analysis for cell viability was performed using one-way ANOVA followed by Tukey’s post hoc test. Time-course Western blot data were analyzed using the Kruskal–Wallis test followed by Dunn’s post hoc test. Statistical significance is indicated as * *p* < 0.05 and ** *p* < 0.01 compared with CON.

To determine optimal conditions for VEGF-induced endothelial cell activation in vitro, we first assessed the impact of VEGF on HUVEC viability (Figure 2A). HUVEC were exposed to graded concentrations of VEGF for 24 h—a duration selected to assess downstream cellular responses elicited by VEGF stimulation. Among the tested concentrations, 5.0 ng/mL VEGF elicited a statistically significant increase in cell viability relative to the control (*p* < 0.01; Figure 2B). On the basis of this dose–response analysis, a VEGF concentration of 5.0 ng/mL was selected for all subsequent experiments. Given that VEGF-induced intracellular signaling precedes downstream cellular responses and exhibits rapid and transient kinetics, we subsequently investigated the temporal dynamics of MAPK signaling pathway activation. Serum-starved HUVEC were stimulated with VEGF, and protein lysates were harvested at early time points ranging from 5 to 60 min (Figure 2C). Western blot analysis demonstrated that VEGF stimulation substantially upregulated phosphorylation of ERK, JNK, and p38 MAPK, while total protein levels remained unchanged (Figure 2D–F).

Quantitative evaluation demonstrated differential activation kinetics among the MAPK family members. Levels of phosphorylated ERK (p-ERK) peaked at 15 min post-VEGF stimulation (*p* < 0.05 compared with CON; Figure 2G). Phosphorylation of JNK significantly increased starting at 15 min post-VEGF stimulation and peaked at 30 min (*p* < 0.05; Figure 2H). In contrast, p38 MAPK phosphorylation significantly increased starting from 10 min post-VEGF stimulation and peaked at 60 min (*p* < 0.05; Figure 2I).

In aggregate, these findings suggest that VEGF increases HUVEC viability and induces rapid activation of multiple MAPK signaling pathways, supporting the suitability of this model for evaluating miRNA-mediated regulation of VEGF-induced endothelial responses.

### 2.3. Immunocytochemical Evidence of Attenuated VEGF-Induced MAPK Phosphorylation in miRNA-Transduced HUVEC

**Figure 3:**
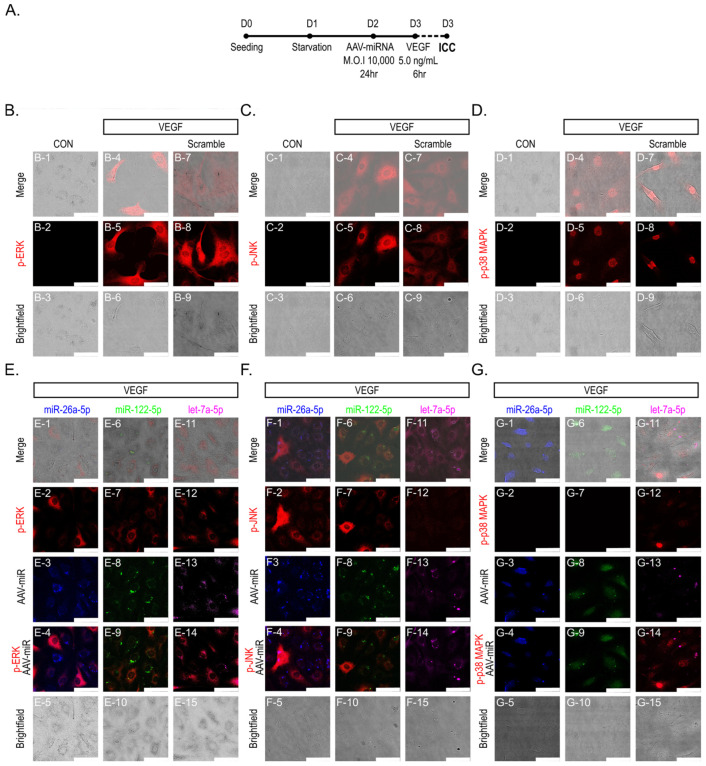
Visualization of miRNA-associated attenuation of VEGF-induced MAPK activation in HUVEC by immunocytochemistry (ICC). (**A**) Experimental timeline for AAV-miRNA transduction, VEGF stimulation, and ICC analysis. D followed by a number indicates the experimental day, and the dashed line represents the duration of VEGF treatment. (**B**–**D**) Representative confocal images of HUVEC stimulated with VEGF (5 ng/mL) for 6 h, showing increased immunofluorescence signals for p-ERK, p-JNK, and p-p38 MAPK compared with control conditions. Comparable signal intensities were observed between VEGF-treated and scramble-transduced cells. (**E**–**G**) Merged images showing that AAV-transduced cells expressing miR-26a-5p (blue, TagBFP+), miR-122-5p (green, EGFP+), or let-7a-5p (magenta, mCherry+) exhibit reduced immunofluorescence signals for phosphorylated MAPKs compared with adjacent non-transduced cells within the same field. Scale bar, 50 μm.

To assess the impact of the three miRNA candidates on VEGF-induced MAPK activation, immunocytochemistry (ICC) was employed to visualize p-ERK, p-JNK, and p-p38 in HUVEC (Figure 3A). Confocal microscopic analysis demonstrated that, under basal conditions, immunofluorescence signals for p-ERK, p-JNK, and p-p38 were negligible in control cells (Figure 3B–D). VEGF stimulation substantially elevated immunofluorescence signals for p-ERK, p-JNK, and p-p38, with comparable intensities observed in both the VEGF-only and scramble-transduced groups. These findings indicate that the AAV vector itself does not influence MAPK activation (Figure 3B–D).

Transduction with miR-26a-5p, miR-122-5p, or let-7a-5p resulted in a reduction in VEGF-induced immunofluorescence signals for p-ERK and p-JNK, with a relatively greater reduction observed in p38 MAPK phosphorylation (Figure 3E–G). Notably, this attenuation was predominantly observed in AAV-transduced cells, whereas adjacent non-transduced cells exhibited persistent robust MAPK activation, as evidenced by the merged images (Figure 3E–G).

### 2.4. Candidate miRNAs Inhibit VEGF-Induced Proliferation and Cell Cycle Progression in HUVEC

**Figure 4:**
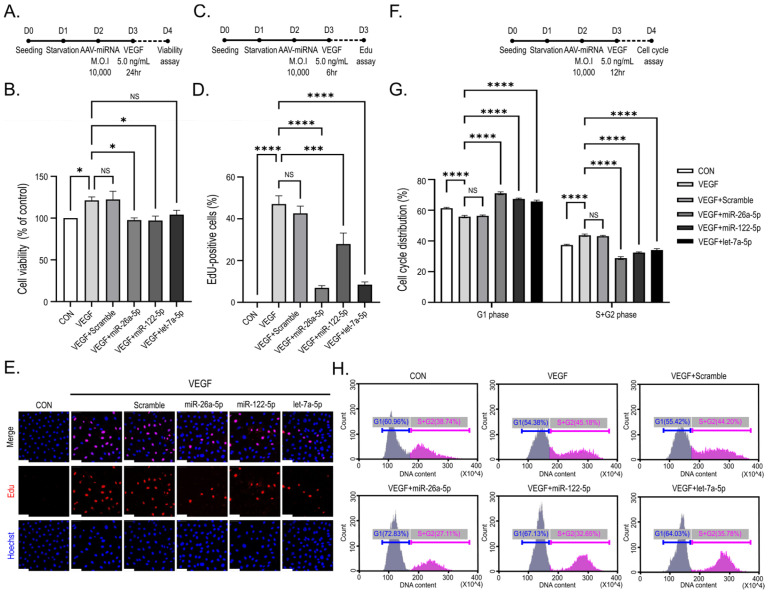
miRNA candidates attenuate VEGF-driven endothelial proliferative responses and promote G1-phase cell cycle arrest. In the experimental schematics (**A**,**C**,**F**), D followed by a number indicates the experimental day, and the dashed lines represent the duration of VEGF treatment. (**A**,**B**) Assessment of cell viability in miRNA-transduced HUVEC 24 h after VEGF stimulation, showing that miRNA transduction did not markedly reduce basal cell viability while attenuating VEGF-induced increases. (**C**–**E**) Representative images and quantification of EdU incorporation, with EdU-positive cells shown in red and Hoechst-stained nuclei shown in blue, at 6 h following VEGF stimulation, indicating reduced DNA synthesis in cells expressing miR-26a-5p, miR-122-5p, or let-7a-5p. For the EdU assay, 3–5 randomly selected fields were analyzed and averaged in each experiment before statistical analysis. (**F**–**H**) Flow cytometric analysis of cell cycle distribution at 12 h post-VEGF stimulation. miRNA expression, particularly miR-26a-5p, was associated with an increased proportion of cells in the G1 phase and a corresponding reduction in S + G2 phases. Data are presented as mean ± SEM from three independent experiments (n = 3). Statistical analysis for cell viability and EdU assays was performed using one-way ANOVA followed by Tukey’s post hoc test. Cell cycle distribution (G1 vs. S + G2) was analyzed using two-way ANOVA with Bonferroni’s post hoc test. Significance is indicated as NS, not significant; * *p* < 0.05, *** *p* < 0.005, and **** *p* < 0.001. Scale bar, 50 μm.

To examine the impact of the three AAV-delivered miRNAs on VEGF-induced endothelial cell proliferation, we evaluated metabolic activity-based cell viability, DNA synthesis, and cell cycle distribution in HUVEC. VEGF stimulation significantly augmented HUVEC viability at 24 h relative to the control (*p* < 0.05; Figure 4B). Transduction with miR-26a-5p and miR-122-5p reduced VEGF-induced HUVEC viability toward control levels (Figure 4A,B). Importantly, transduction with all three miRNAs did not markedly reduce cell viability, indicating that the observed changes were not attributable to cytotoxic effects.

To evaluate early proliferative responses, an EdU incorporation assay was conducted 6 h post-VEGF stimulation. Quantitative analysis revealed that transduction with all three miRNA candidates significantly attenuated the proportion of EdU-positive cells relative to VEGF-stimulated control, indicating reduced DNA synthesis (*p* < 0.001; Figure 4C–E).

To investigate whether the observed reduction in DNA synthesis corresponded to alterations in cell cycle progression, flow cytometric analysis of cell cycle distribution was performed 12 h post-VEGF treatment. VEGF stimulation elevated the proportion of cells in the S + G2 phases, whereas transduction with each of the three miRNAs was associated with an increased proportion of cells in the G1 phase and a corresponding reduction in S + G2 populations (*p* < 0.001; Figure 4F,G). In particular, transduction with miR-26a-5p resulted in a higher proportion of cells in the G1 phase (72.83%) (Figure 4H).

Taken together, these findings indicate that miR-26a-5p, miR-122-5p, and let-7a-5p attenuate VEGF-induced endothelial proliferation through suppression of DNA synthesis and an increase in G1-phase cell cycle population, without eliciting overt cytotoxicity.

### 2.5. miR-122-5p Inhibits VEGF-Induced HUVEC Migration

**Figure 5:**
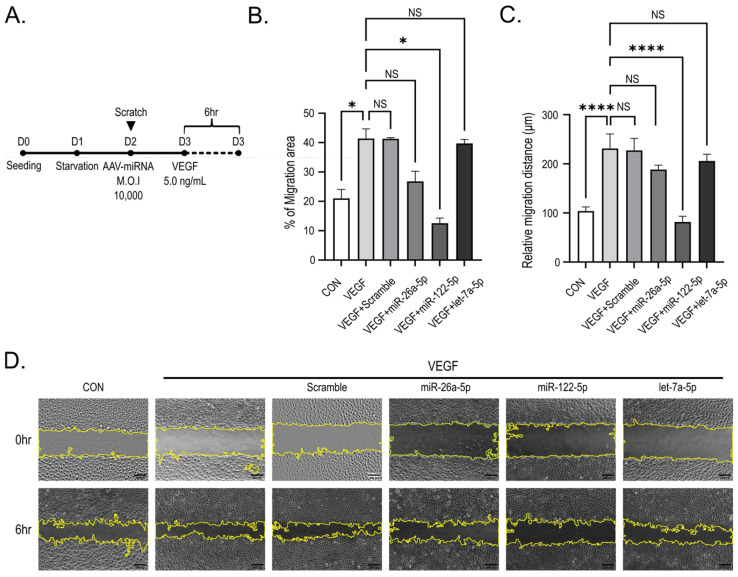
miR-122-5p attenuates VEGF-induced endothelial cell migration. (**A**) Experimental schematic for the scratch assay. D followed by a number indicates the experimental day, and the dashed line represents the duration of VEGF treatment. (**B**,**C**) Quantification of migration area (%) and relative migration distance (μm) following VEGF stimulation (5 ng/mL) for 6 h, analyzed using the MRI Wound Healing Tool in ImageJ (version 1.54f; NIH, Bethesda, MD, USA). For the scratch assay, 3–5 randomly selected fields were analyzed and averaged in each experiment before statistical analysis. (**D**) Representative phase-contrast images of scratch wounds at 0 and 6 h after VEGF stimulation in control and miRNA-transduced HUVEC, showing differential wound closure. Yellow lines indicate the wound edges automatically delineated by the MRI Wound Healing Tool in ImageJ. Data are presented as mean ± SEM from three independent experiments (n = 3). Statistical significance was determined using one-way ANOVA followed by Tukey’s post hoc test. Significance is indicated as NS, not significant; * *p* < 0.05, and **** *p* < 0.001 compared with the VEGF-treated group. Scale bar, 200 μm.

To assess the impact of the three AAV-delivered miRNA candidates on VEGF-induced endothelial cell migration, we conducted a scratch assay (Figure 5A). VEGF treatment significantly increased both the migration area and the relative migration distance in HUVEC (*p* < 0.05 and *p* < 0.001, respectively; Figure 5B,C).

Of the three miRNA candidates, miR-122-5p was the only miRNA that significantly attenuating both the migration area and relative migration distance, reducing these parameters toward levels observed in the untreated control (*p* < 0.05 and *p* < 0.001, respectively; Figure 5B,C). In contrast, transduction with miR-26a-5p or let-7a-5p resulted in only modest reductions in migratory parameters, which failed to achieve statistical significance under the experimental conditions employed (NS; Figure 5B,C).

Representative micrographs obtained 6 h post-scratch formation were consistent with these quantitative findings, demonstrating that VEGF stimulation elicited marked constriction of the scratch gap, whereas the gap in miR-122-5p-transduced cells remained relatively preserved, closely resembling the untreated control (Figure 5D). Collectively, these findings suggest that miR-122-5p acts as an inhibitor of VEGF-induced endothelial cell migration.

### 2.6. Candidate miRNAs Suppress VEGF-Induced Capillary-like Network Formation in HUVEC

**Figure 6:**
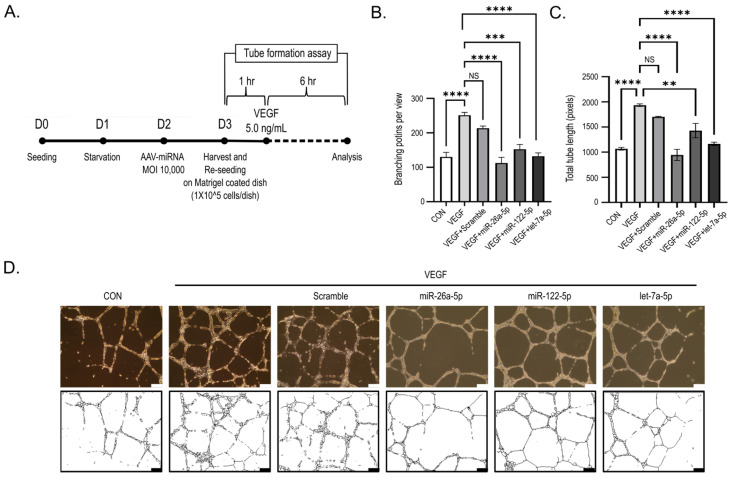
miRNA candidates attenuate VEGF-induced capillary-like tube formation in HUVEC. (**A**) Experimental schematic for the Matrigel-based tube formation assay following AAV-miRNA transduction and VEGF stimulation. D followed by a number indicates the experimental day, and the dashed line represents the duration of VEGF treatment. (**B**,**C**) Quantitative analysis of tube formation, quantified as the number of branching points per view and total tube length (pixels), analyzed using an ImageJ-based Angiogenesis Analyzer macro. For the tube formation assay, 3–5 randomly selected fields were analyzed and averaged in each experiment before statistical analysis. (**D**) Representative phase-contrast images and corresponding skeletonized images of capillary-like tube networks formed on growth factor-reduced Matrigel after VEGF stimulation (5 ng/mL) for 6 h in control and miRNA-transduced HUVEC. Data are presented as mean ± SEM from three independent experiments (n = 3). Statistical significance was determined using one-way ANOVA followed by Tukey’s post hoc test. Significance is indicated as NS, not significant; ** *p* < 0.01, *** *p* < 0.005, and **** *p* < 0.001 compared with the VEGF-treated group. Scale bar, 500 μm.

To assess the impact of the three AAV-delivered miRNA candidates on VEGF-induced endothelial tubulogenesis and capillary-like network formation, HUVEC were subjected to a Matrigel-based tube formation assay (Figure 6A). VEGF stimulation significantly augmented both the number of branching points and the total tube length relative to the untreated control (*p* < 0.001; Figure 6B,C).

In contrast, transduction with the three candidate miRNAs significantly reduced VEGF-induced tube formation. Notably, miR-26a-5p showed a greater inhibitory effect, significantly reducing both the number of branching points and total tube length (*p* < 0.001; Figure 6B,C). miR-122-5p and let-7a-5p likewise significantly inhibited VEGF-induced tube formation, as indicated by reductions in the number of branching points and total tube length (*p* < 0.01–0.001, depending on the parameter), albeit to a lesser extent than miR-26a-5p (Figure 6B,C).

Representative phase-contrast micrographs corroborated the quantitative findings. In contrast to the elaborate, interconnected capillary-like networks induced by VEGF stimulation, HUVEC transduced with the candidate miRNAs displayed disrupted tubular architectures, characterized by fragmented segments and incomplete structures (Figure 6D).

Collectively, these findings suggest that the candidate miRNAs significantly suppress VEGF-induced capillary-like network formation in HUVEC.

## 3. Discussion

### 3.1. Implications of MAPK Signaling Modulation in the Pathogenesis of Retinal Diseases

Pathological retinal neovascularization constitutes a primary cause of vision loss in nAMD and DR [32,33,34]. Although anti-VEGF therapies demonstrate clinical efficacy, their limited intraocular half-life necessitates repeated intravitreal injections, thereby compromising long-term therapeutic durability [35,36]. Prior investigations have demonstrated that exosomes derived from day-60 retinal organoids are enriched in miR-26a-5p, miR-122-5p, and let-7a-5p, exerting neuroprotective effects through modulation of MAPK signaling pathways [18]. The present study extends these prior observations by establishing that this specific miRNA trio (miR-26a-5p, miR-122-5p, and let-7a-5p) potently suppresses VEGF-induced activation of MAPK signaling pathways in HUVEC.

These findings indicate that MAPK signaling serves as a convergent pathological nexus across diverse ocular cell types implicated in retinal disease pathogenesis, including photoreceptors and vascular endothelial cells [33,34,35,36,37,38]. miRNAs derived from early-stage retinal organoids thus mediate pleiotropic regulatory effects predominantly through convergent signaling pathways, rather than via strictly cell type-specific mechanisms [18,39,40]. This pathway-level regulation holds substantial therapeutic potential for complex retinal disorders characterized by the coexistence of neurodegeneration and pathological angiogenesis.

We previously validated the therapeutic impact of RO-EXO obtained from retinal organoids in a mouse CNV model. RO-EXO demonstrated a therapeutic effect in a model of laser-induced CNV through intravitreal injection. Moreover, exosome-derived miRNA were validated to provide therapeutic benefits, including reducing lesion size and vascular area in the in vivo CNV model. Nevertheless, it should be noted that HUVEC represent large-vessel endothelial cells and may not fully recapitulate the specific characteristics of retinal microvascular endothelial cells.

### 3.2. MAPK Signaling: A Central Convergence Node in VEGF-Induced Angiogenesis

VEGF-induced angiogenesis is initiated through the activation of MAPK signaling pathways, which govern critical endothelial cell functions, including proliferation, migration, and structural reorganization [41,42,43]. In the present study, VEGF-A165—the predominant and most bioactive isoform of VEGF—was employed to elicit angiogenic signaling in endothelial cells, given its well-documented role in activating VEGFR2-dependent MAPK signaling cascades [12,44]. Temporal phosphoproteomic analyses substantiated that VEGF stimulation elicits distinct phosphorylation kinetics of ERK, JNK, and p38 MAPK in HUVEC, thereby corroborating the implication of MAPK signaling in the incipient endothelial responses (Figure 2D–I) [11,45]. Immunocytochemical analyses conducted at single-cell resolution demonstrated that miRNA-mediated attenuation of MAPK phosphorylation was restricted to AAV-transduced cells, whereas adjacent non-transduced cells exhibited sustained robust activation (Figure 3). This spatially restricted modulation confirms the cell-intrinsic nature of the observed effects, which are directly attributable to miRNA expression rather than arising from non-specific extracellular influences.

### 3.3. Inhibition Across Multiple Stages of Angiogenesis

Angiogenesis entails a series of sequential stages encompassing endothelial cell activation, proliferation, migration, and tube formation [46]. AAV-mediated delivery of the candidate miRNAs elicited suppressive effects across multiple stages of angiogenesis, rather than being confined to a single functional parameter. All three miRNAs significantly inhibited DNA synthesis and elicited G1-phase cell cycle arrest, thereby suppressing endothelial proliferative activity (Figure 4)

The absence of significant cytotoxicity indicates that the observed reductions in metabolic activity and proliferation are unlikely attributable to non-specific cell death. Subsequent functional assays demonstrated differential inhibitory effects among the candidate miRNAs on distinct VEGF-induced angiogenic processes: miR-122-5p predominantly suppressed endothelial cell migration (Figure 5), whereas miR-26a-5p more potently inhibited proliferative capacity and capillary-like network formation (Figure 4 and Figure 6). These findings indicate that each miRNA preferentially modulates distinct facets of angiogenic processes through partially overlapping regulatory pathways, thereby conferring a synergistic inhibitory effect.

### 3.4. Validation of Underlying Mechanisms Through GO Enrichment Analysis

GO enrichment analysis of the experimentally validated target genes retrieved from miRTarBase for this miRNA trio revealed significant overrepresentation of biological processes associated with the negative regulation of angiogenesis and MAPK signaling, thereby providing bioinformatics support for their anti-angiogenic potential (Figure 1H–M). These bioinformatics results furnish a mechanistic rationale underpinning our experimental functional observations. Notably, the pronounced enrichment of GO terms pertaining to “regulation of the G1/S transition” for miR-26a-5p and “regulation of endothelial cell migration” for miR-122-5p accords with the findings from our functional assays (Figure 1, Figure 4 and Figure 5).

Furthermore, the uniform enrichment of “protein kinase activity” and “binding” across all three miRNAs aligns with the suppression of the MAPK signaling axis observed in our analyses (Figure 2 and Figure 3). As miRNAs act primarily as post-transcriptional repressors of translation, the observed GO enrichment profiles indicate a coordinated downregulation of critical signaling components essential for VEGF-mediated angiogenesis [47,48]. These findings align with previous investigations, which identify miR-26a-5p as an anti-angiogenic suppressor through targeting of VEGFA and MAPK6 [49], miR-122-5p as an inhibitor of VEGF-mediated signaling [50], and the let-7 family as a repressor of pathological ocular angiogenesis, notwithstanding its multifaceted role in DR [51].

### 3.5. Stimulus-Dependent miRNA Regulatory Profiles and Therapeutic Implications

Although these miRNAs exert consistent anti-angiogenic effects in VEGF-dominant contexts, paradoxical pro-angiogenic roles have been documented in alternative biological settings miR-26a-5p and let-7 family members have been implicated in pro-angiogenic signaling within ischemic or tissue repair models, wherein alternative angiogenic cascades—such as those orchestrated by hypoxia-inducible factors amid diminished VEGF abundance—predominate [23,52]. This context-dependent functional divergence underscores that miRNA bioactivity is not an invariant attribute but is contingent upon the prevailing signaling milieu.

### 3.6. Utilizing the AAV2.7m8 Platform for Targeted miRNA-Mediated Interventions

The AAV2.7m8 system enables stable, cell-intrinsic expression, thereby circumventing the pharmacokinetic and manufacturing limitations inherent to native exosomes. Importantly, the concurrent suppression of ERK, JNK, and p38 MAPK furnishes a mechanistic rationale for the anti-angiogenic efficacy previously documented in laser-induced CNV models [19].

The present study demonstrates that AAV-mediated delivery of miR-26a, miR-122, and let-7a effectively suppresses VEGF-induced angiogenic responses in endothelial cells. These findings highlight the potential of miRNA-based strategies to complement or extend current anti-VEGF therapies. Unlike direct VEGF inhibition, which targets a single ligand-receptor axis, miRNAs exert pleiotropic regulatory effects across multiple signaling pathways, including MAPK, thereby offering broader control over endothelial activation. This convergence on MAPK signaling suggests that miRNAs may provide a more durable and multifaceted suppression of pathological angiogenesis. Importantly, the context-dependent roles of these miRNAs—pro-angiogenic in certain tissues yet anti-angiogenic in the retinal environment—underscore the need to consider cellular context when designing therapeutic applications. Together, these results position AAV-delivered miRNAs as a promising alternative to exosome-based delivery, overcoming the instability of vesicle-mediated transfer while enabling sustained expression in target cells.

Despite these findings, several limitations of the present study merit acknowledgment. First, while HUVECs are a well-established in vitro model for endothelial research, they may not fully recapitulate the phenotypic and functional characteristics of the retinal microvascular endothelium. Future studies employing retinal-specific endothelial cells would provide greater translational relevance to retinal vascular pathophysiology. Second, our experimental system focused on VEGF-driven angiogenic responses under standardized conditions, and thus did not incorporate complex microenvironmental factors such as hyperglycemia or chronic inflammation, which are prevalent in vasoproliferative retinopathies. Finally, although the experiments were independently repeated three times, the limited sample size and the inherent variability of functional assays, such as migration and tube formation, may reduce statistical power. Larger-scale studies will therefore be needed to further strengthen the robustness of these findings. In addition, potential crosstalk with other signaling pathways remains to be elucidated.

## 4. Materials and Methods

### 4.1. Cell Culture

Human umbilical vein endothelial cells (HUVEC; Catalog No. C2519A, Lonza, Walkersville, MD, USA) were cultured in Endothelial Cell Growth Medium-2 BulletKit (Catalog No. CC-3162, Lonza, Walkersville, MD, USA). The complete medium was formulated by supplementing EBM-2 Basal Medium (Catalog No. CC-3156, Lonza, Walkersville, MD, USA) with the EGM-2 SingleQuots Supplement Pack (Catalog No. CC-4176, Lonza, Walkersville, MD, USA), comprising hydrocortisone, human fibroblast growth factor-B, vascular endothelial growth factor, R3-insulin-like growth factor-1, ascorbic acid, human epidermal growth factor, gentamicin/amphotericin-B, heparin, and 2% fetal bovine serum. HUVEC were maintained at 37 °C in a humidified atmosphere containing 5% CO_2_. Cells between passages 3 and 7 were used for all experiments.

### 4.2. AAV2.7m8 Vector Construction and Transduction

Recombinant AAV2.7m8 vectors expressing the candidate miRNAs were generated by VectorBuilder (Chicago, IL, USA). The miRNA expression cassettes were placed under the control of a U6 promoter and packaged within AAV2.7m8 capsids, with each miRNA co-expressed alongside a distinct fluorescent reporter to facilitate assessment of transduction efficiency: TagBFP for miR-26a-5p, EGFP for miR-122-5p, and mCherry for let-7a-5p. Prior to AAV transduction, HUVEC were serum-starved for 12 h in EBM-2 basal medium to synchronize cells in the G0/G1 phase and minimize basal VEGF and MAPK signaling. Transduction was performed at a multiplicity of infection of 10,000 genome copies (GC)/cell, based on the viral titer (GC/mL) provided by the manufacturer.

Transduction efficiency was quantified 24 h post-AAV transduction via flow cytometry using a DxFLEX flow cytometer (Beckman Coulter, Brea, CA, USA) and was defined as the percentage of fluorescent reporter-positive cells within the singlet population. The fluorescence threshold was established using corresponding non-transduced controls (WO). Data acquisition and analysis were conducted using CytExpert software (version 2.3.5.284; Beckman Coulter, Brea, CA, USA). Events with low forward scatter were first excluded to remove small debris. The main cell population was identified using FSC-A versus side scatter (SSC-A) plots, and singlets were strictly gated based on the correlation between FSC-A and FSC-height (FSC-H) to exclude doublets. Only verified singlet events were further analyzed in the TagBFP, EGFP, and mCherry channels. All experiments were performed in three independent experiments. The nucleotide sequences of all miRNA inserts are provided in Supplementary Table S1.

### 4.3. VEGF Stimulation

Following 24 h of AAV transduction, HUVEC were stimulated with recombinant human vascular endothelial growth factor 165 (rhVEGF_165_; Catalog No. 293-VE/CF, R&D Systems, Minneapolis, MN, USA) at a concentration of 5.0 ng/mL, which was selected based on the dose–response analysis. The duration of VEGF stimulation was varied according to the requirements of the respective functional assay.

### 4.4. Bioinformatic Analysis of miRNA Targets and Functional Enrichment

The experimentally validated target genes for the human miRNAs miR-26a-5p, miR-122-5p, and let-7a-5p were retrieved from miRTarBase (Release 10.0; https://mirtarbase.cuhk.edu.cn/, downloaded on 19 September 2025). These target genes were obtained from a publicly available database and were not derived from gene expression data generated in the present study. All subsequent analyses were conducted in silico. Functional enrichment analysis of these database-derived target genes was performed using the Enrichr platform (https://maayanlab.cloud/Enrichr/, accessed on 19 September 2025), with an emphasis on GO terms pertaining to Biological Processes (BP) and Molecular Functions (MF). The significance of the enrichments was determined using adjusted *p*-values, with results visualized on a—log_10_ scale.

### 4.5. Cell Viability Assay

Cell viability of HUVEC was evaluated using the EZ-CYTOX Cell Viability, Proliferation & Cytotoxicity Assay Kit (Daeil Lab Service, Seoul, Republic of Korea), a colorimetric assay based on cellular metabolic activity. HUVEC were plated in 96-well plates at a density of 1 × 10^4^ cells/well and subjected to serum starvation for 12 h in EBM-2 medium. For VEGF dose–response experiments, HUVEC were stimulated with recombinant human VEGF at concentrations ranging from 0.1 to 20.0 ng/mL for 24 h. For AAV-miRNA experiments, HUVEC were serum-starved prior to transduction with AAV2.7m8-miRNA vectors for 24 h, followed by stimulation with 5.0 ng/mL VEGF for 24 h. Following the respective treatments, 10 μL of EZ-CYTOX reagent was added to each well, and the plates were incubated at 37 °C for 2 h. Absorbance was subsequently quantified at 450 nm using a microplate spectrophotometer. Cell viability was determined according to the following formula:

(1) $$Cell\;viability\;\left(\%\right)=\frac{Aexp-Ablank}{Acon-Ablank}\times 100$$

where Aexp denotes the absorbance of VEGF-treated wells (including VEGF-only, VEGF + Scramble, and VEGF + miRNA groups), Acon denotes the absorbance of non-transduced, non-VEGF-treated baseline control wells, and Ablank denotes the absorbance of blank wells containing medium without cells. Cell viability assay was performed in three independent experiments, with three wells per condition in each experiment.

### 4.6. Western Blot

HUVEC were seeded and cultured until reaching approximately 70% confluence. Cells were then serum-starved for 12 h in EBM-2 medium, followed by stimulation with 5.0 ng/mL recombinant human VEGF for 0, 5, 10, 15, 30, or 60 min. Following stimulation, cells were rinsed with ice-cold phosphate-buffered saline (PBS) and lysed in radioimmunoprecipitation assay buffer supplemented with protease and phosphatase inhibitor cocktails (GenDEPOT, Barker, TX, USA). Protein concentrations were quantified using the bicinchoninic acid assay (Thermo Fisher Scientific, Waltham, MA, USA). Equal amounts of protein (10 μg) were resolved by sodium dodecyl sulfate–polyacrylamide gel electrophoresis and electrotransferred to polyvinylidene difluoride membranes (Millipore, Burlington, MA, USA).

Subsequently, the membranes were blocked with 5% (*w*/*v*) bovine serum albumin in PBST (phosphate-buffered saline containing 0.1% Tween-20) for 1 h at room temperature, followed by overnight incubation at 4 °C with primary antibodies targeting the total and phosphorylated forms of ERK1/2, JNK, and p38 MAPK (Cell Signaling Technology, Danvers, MA, USA). Following washes, the membranes were incubated with horseradish peroxidase-conjugated secondary antibodies (GenDEPOT, Barker, TX, USA). Protein bands were visualized using a chemiluminescent substrate (EzWestLumi Plus; ATTO, Tokyo, Japan).

The intensity of immunoreactive bands was quantified via densitometric analysis using ImageJ software (version 1.54f; NIH, Bethesda, MD, USA). The relative levels of protein phosphorylation were quantified by normalizing the densitometric intensities of phosphorylated protein bands to those of their corresponding total protein bands. Western blot analyses were performed in three independent experiments.

### 4.7. Immunocytochemistry

HUVEC were seeded onto 8-well chamber slides (SPL Life Sciences, Pocheon, Republic of Korea) at a density of 2 × 10^4^ cells per well. HUVEC were serum-starved for 12 h in EBM-2 basal medium and subsequently transduced with AAV2.7m8-miRNA vectors at a multiplicity of infection of 10,000 for 24 h. Following transduction, cells were stimulated with 5.0 ng/mL VEGF for 6 h.

Following treatment, cells were fixed in 4% paraformaldehyde prepared in PBS for 15 min at room temperature, followed by three washes with PBS. Cells were subsequently permeabilized with 0.1% Triton X-100 in PBS for 10 min, followed by blocking with 5% bovine serum albumin (BSA) in PBS for 60 min at room temperature. Cells were subsequently incubated with primary antibodies targeting the phosphorylated forms of ERK1/2, JNK, and p38 MAPK (1:200; Cell Signaling Technology, Danvers, MA, USA) for 2 h at room temperature. Following washes, cells were incubated with species-specific secondary antibodies conjugated to Alexa Fluor 647 (1:1000 dilution; Invitrogen, Carlsbad, CA, USA) for 2 h at room temperature, protected from light.

The chambers were removed, and the slides were mounted using an anti-fade fluorescence mounting medium (Abcam, Cambridge, UK). Confocal images were obtained using a Leica TCS SP8 confocal microscope (Leica Microsystems, Wetzlar, Germany).

### 4.8. EdU Proliferation Assay

HUVEC were seeded in glass-bottom confocal dishes at a density of 5 × 10^3^ cells per dish. A reduced seeding density was employed to achieve sufficient spatial separation between individual cells, thereby enabling precise quantification of EdU incorporation. Following serum starvation for 12 h in EBM-2 medium, cells were transduced with AAV-miRNA vectors at a multiplicity of infection of 10,000 for 24 h, followed by stimulation with 5.0 ng/mL VEGF for 6 h. EdU (10 μM) was added to the cultures during the final 2 h of VEGF stimulation.

The Click-iT EdU Cell Proliferation Kit (Alexa Fluor™ 647; Thermo Fisher Scientific, Waltham, MA, USA) was employed for EdU detection, cell permeabilization, and nuclear counterstaining with Hoechst 33342 (Invitrogen, Carlsbad, CA, USA), in strict accordance with the manufacturer’s protocol. Coverslips were mounted in an anti-fade fluorescence mounting medium (Abcam, Cambridge, UK), and confocal images were acquired using a Leica TCS SP8 confocal microscope (Leica Microsystems, Wetzlar, Germany). The percentage of EdU-positive cells relative to total Hoechst-positive nuclei was quantified using ImageJ software (version 1.54f; NIH, Bethesda, MD, USA). All EdU proliferation assays were performed in three independent biological replicates. EdU proliferation assay was performed in three independent experiments. For each sample, at least five randomly selected fields were analyzed and averaged before statistical analysis.

### 4.9. Flow Cytometry-Based Analysis of Cell Cycle Distribution

To assess cell cycle distribution, HUVEC were synchronized by serum starvation for 12 h, followed by transduction with AAV-miRNA vectors at a multiplicity of infection of 10,000 for 24 h and subsequent stimulation with 5.0 ng/mL VEGF for 12 h. Cells were harvested and fixed in BD Phosflow™ Fix Buffer I (BD Biosciences, San Jose, CA, USA) for 20 min, followed by permeabilization in BD Phosflow™ Perm/Wash Buffer I (BD Biosciences, San Jose, CA, USA). To prevent spectral overlap with fluorescent reporters, DNA content was labeled using FxCycle™ Far Red Stain (Thermo Fisher Scientific, Waltham, MA, USA) in the presence of RNase A for 30 min.

To ensure data fidelity and exclude cell aggregates or doublets, only singlet events were gated and analyzed using forward scatter area versus height/width and side scatter area versus height/width parameters prior to fluorescence quantification. Data acquisition was performed using a DxFLEX flow cytometer (Catalog No. C54329AB; Beckman Coulter, Brea, CA, USA), followed by analysis with CytExpert software (version 2.3.5.284; Beckman Coulter, Brea, CA, USA). At least 10,000 events were acquired per sample, and the proportions of cells in the G1, S + G2 phases were quantified from the resulting DNA content histograms. Flow cytometric analyses were performed in three independent experiments.

### 4.10. Scratch Assay

HUVEC were seeded into 35 mm confocal dishes and cultured until reaching near-confluence. HUVEC were serum-starved for 12 h and subsequently transduced with AAV-miRNA vectors at a multiplicity of infection of 10,000 for 24 h. A linear scratch was generated across the confluent monolayer using a sterile cell scraper, followed by gentle washing with PBS to remove non-adherent cells and establish a uniform acellular scratch area. Subsequently, cells were stimulated with 5.0 ng/mL VEGF for 6 h.

Images were acquired at 0 h and 6 h after VEGF stimulation using an EVOS inverted microscope (Thermo Fisher Scientific, Waltham, MA, USA). Migration was quantified using the ImageJ MRI Wound Healing Tool macro (version 1.54f; NIH, Bethesda, MD, USA). The ImageJ MRI Wound Healing Tool macro was utilized to automatically delineate the acellular wound area and compute the following parameters:

(2) $$1.\;Migration\;area\;\left(\%\right)=\frac{Area0h-Area6h}{Area0h}\times 100$$

(3) $$2.\;Relative\;migration\;distance\;\left(\mu m\right)=\frac{Width0h-Width6h}{2}$$

where Area0h and Area6h denote the initial and final scratch areas, respectively, and Width0h and Width6h represent the corresponding initial and final scratch widths. All scratch assays were performed in three independent experiments. Scratch assays were performed in three independent experiments. For each sample, migration parameters were quantified using 3–5 randomly selected fields of view and averaged before statistical analysis.

### 4.11. Tube Formation Assay

HUVEC were seeded into 35 mm dishes and cultured until reaching near-confluence. Cells were serum-starved for 12 h and subsequently transduced with AAV-miRNA vectors at a multiplicity of infection of 10,000 for 24 h. Following transduction, cells were harvested into a single-cell suspension and re-plated at a density of 80,000 cells/cm^2^ onto 35 mm dishes pre-coated with growth factor-reduced Matrigel (Cat. No. 354230; Corning, Glendale, AZ, USA). Prior to cell seeding, Matrigel-coated dishes were polymerized at 37 °C. One hour following re-seeding, recombinant human VEGF was added to the cultures at 5.0 ng/mL to stimulate capillary-like tube formation for 6 h.

Capillary-like structures were imaged using an EVOS inverted microscope (Thermo Fisher Scientific, Waltham, MA, USA). Tube formation was quantified using ImageJ software (Fiji, v1.54f; NIH, Bethesda, MD, USA) employing a macro (Angiogenesis_Analyzer.ijm). For each sample, 3–5 randomly selected fields of view were captured, and all parameters were averaged before statistical analysis. The following parameters were quantified:

1. Number of branching points, defined as junctions where at least three tubes intersect, serving as an indicator of network complexity.
2. Total tube length, measured as the cumulative length of tubular structures (in pixels). Tube formation assays were performed in three independent experiments.

### 4.12. Statistical Analysis

Data are presented as the mean ± standard error of the mean from at least three independent experiments. Statistical analyses were performed using GraphPad Prism (version 10.0; GraphPad Software, San Diego, CA, USA) and IBM SPSS Statistics software (version 22.0; IBM Corp., Armonk, NY, USA). All statistical analyses were performed using two-tailed tests.

1. Comparisons between two groups were conducted using an unpaired Student’s *t*-test.
2. Multiple-group comparisons in the cell viability, EdU proliferation, scratch, and tube formation assays were conducted using one-way analysis of variance followed by Tukey’s post hoc test.
3. Western blot data were analyzed using the non-parametric Kruskal–Wallis test followed by Dunn’s post hoc test.
4. Cell cycle distribution (G1 vs. S + G2 phases) was analyzed using two-way analysis of variance followed by Bonferroni post hoc test.

Statistical significance was defined as *p* < 0.05. Levels of significance are indicated as follows: * *p* < 0.05, ** *p* < 0.01, *** *p* < 0.005, and **** *p* < 0.001. Non-significant differences are indicated as ‘NS’.

## 5. Conclusions

In summary, the current study demonstrates that AAV-mediated delivery of a defined miRNA triplet potently suppresses VEGF-stimulated angiogenesis through concerted modulation of pivotal signaling nodes. Our experimental findings, including G1-phase cell cycle arrest and attenuated endothelial migratory capacity, corroborate with the bioinformatic predictions of miRNA functional targets. Focusing on the MAPK signaling pathway, this multi-target miRNA approach provides a strong justification for enduring gene therapy, likely alleviating the drawbacks associated with existing single-target anti-VEGF treatments.

## Data Availability

The data presented in this study are available on request from the corresponding author.

## Supplementary Materials

The following supporting information can be downloaded at: https://www.mdpi.com/article/10.3390/ijms27073123/s1.

## Abbreviations

The following abbreviations are used in this manuscript:

nAMD	neovascular age-related macular degeneration
DR	diabetic retinopathy
HUVEC	Human umbilical vein endothelial cells
VEGF-A	vascular endothelial growth factor A
VEGF	vascular endothelial growth factor
MAPK	mitogen-activated protein kinase
ERK	extracellular signal-regulated kinase
JNK	c-Jun N-terminal kinase
EV	Extracellular vesicles
miRNA	microRNAs
RO-Exo	retinal organoid-derived exosomes
CNV	choroidal neovascularization
RPE	retinal pigment epithelium
GO	Gene Ontology
ICC	immunocytochemistry
p-ERK	phosphorylated ERK
p-JNK	Phosphorylated JNK
p-p38	Phosphorylated p38
BP	Biological Processes
MF	Molecular Functions
PBS	phosphate-buffered saline
