# Supplementary material for: AAV2.7m8-Mediated MicroRNA Expression Suppresses VEGF-Induced Angiogenic Responses in HUVEC
Source: Int J Mol Sci. 2026 Mar 30;27(7):3123. doi: 10.3390/ijms27073123 (PMC13072725; doi:10.3390/ijms27073123)
Supplement: Supplementary file 1 [file ijms-27-03123-s001.zip › Supplementary Table S1 legend.pdf]

Supplementary Table S1. Sequences of miRNA candidates used in this study.
