# Supplementary material for: AAV2.7m8-Mediated MicroRNA Expression Suppresses VEGF-Induced Angiogenic Responses in HUVEC
Source: Int J Mol Sci. 2026 Mar 30;27(7):3123. doi: 10.3390/ijms27073123 (PMC13072725; doi:10.3390/ijms27073123)
Supplement: Supplementary file 1 [file ijms-27-03123-s001.zip › Supplementary table S1.pdf]

Supplementary Table S1.

A.

miRNA	miRNA sequences (5' → 3')
hsa-mir-122-5p	uggagugugacaaugguguuug
hsa-let-7a-5p	ugagguaguagguuguauaguu
hsa-mir-26a-5p	uucaaguaauccaggauaggcu
